# PTEN loss of expression predicts cetuximab efficacy in metastatic colorectal cancer patients

**DOI:** 10.1038/sj.bjc.6604009

**Published:** 2007-10-16

**Authors:** M Frattini, P Saletti, E Romagnani, V Martin, F Molinari, M Ghisletta, A Camponovo, L L Etienne, F Cavalli, L Mazzucchelli

**Affiliations:** 1 Institute of Pathology, via in Selva 24, CH-6600 Locarno, Switzerland; 2 Oncology Institute of Southern Switzerland, Ospedale San Giovanni, CH-6500 Bellinzona, Switzerland

**Keywords:** colorectal cancer, cetuximab, EGFR, K-Ras, PTEN, fluorescent *in situ* hybridisation

## Abstract

To evaluate whether the epidermal growth factor receptor (EGFR), K-Ras and PTEN, all members of the EGFR signalling pathway, may affect the clinical response in cetuximab-treated metastatic colorectal cancer (mCRC) patients. Twenty-seven cetuximab-treated mCRC patients were evaluated for drug response and investigated for EGFR protein expression and gene status, *K-Ras* mutational status and PTEN protein expression. Ten patients achieved a partial response (PR) to cetuximab-based therapy. All 27 patients showed EGFR protein overexpression. Epidermal growth factor receptor gene amplification was observed in eight out of 27 (30%) and chromosome 7 marked polysomy in 16 (59%) patients. Partial response was observed in six out of eight patients with *EGFR* gene amplification, four out of 16 with marked polysomy and none out of three with eusomy (*P*<0.05). The *K-Ras* wild-type sequence was observed in 17 patients, and nine of them experienced a PR. Conversely, *K-Ras* was mutated in 10 cases, of which one patient experienced a PR (*P*<0.05). The PTEN protein was normally expressed in 16 patients, and 10 of them achieved a PR. In contrast, no benefit was documented in 11 patients with loss of PTEN activity (*P*<0.001). Patients with *EGFR* gene amplification or chromosome 7 marked polysomy respond to cetuximab. In addition to *K-Ras* mutations, we demonstrate for the first time that the loss of PTEN protein expression is associated with nonresponsiveness to cetuximab.

Metastatic colorectal cancer (mCRC) is a leading cause of cancer death worldwide, and despite recent advances in chemotherapeutic treatment, there is a continuous need for more effective therapies. More recently, specific molecular processes have been targeted for therapeutic interventions. The epidermal growth factor receptor (EGFR) is one of four HER-family tyrosine kinases (EGFR, erbB2, erbB3, erbB4) that initiates intracellular proliferation signalling. The activation results in proliferation and survival through the Ras/Raf/MEK/ERK or PI3K/PTEN/AKT pathways, respectively (Baselga, 2001). The activated EGFR also regulates the production of angiogenic factors and permits tumour invasion through extracellular matrix components. In mCRC, the expression of EGFR, which can be demonstrated in approximately 70% of cases, correlates with poor prognosis (Mayer *et al*, 1993). Given the myriad of downstream effects, its frequency of overexpression, and its correlation with prognosis, various approaches have been considered to inhibit EGFR, including monoclonal antibodies (MoAb) and small molecule inhibitors. In mCRC, the clinical development focused on cetuximab, a chimaeric mouse/human MoAb of the IgG1 subclass, that binds to the extracellular region of the receptor and functions as a competitive antagonist that inhibits ligand binding, leading to the blockage of EGFR downstream pathway.

Several clinical trials including cetuximab have been conducted in mCRC, not only in patients refractory to irinotecan-based chemotherapy, but also as single agent or as first- or second line in combination with oxaliplatin-based regimens. All these studies indicated that only a subgroup of patients treated with cetuximab may benefit from the drug (Rosenberg *et al*, 2002; Van Laethem *et al*, 2003; Cunningham *et al*, 2004; Saltz *et al*, 2001, 2004; Tabernero *et al*, 2004; Jennis *et al*, 2005; Borner *et al*, 2006; Folprecht *et al*, 2006; Venook *et al*, 2006; Wilke *et al*, 2006). Based on preclinical findings, it should be outlined that the trials including cetuximab have been performed in patients who expressed EGFR protein in primary tumours based on immunohistochemistry (IHC). However, no correlation has been shown between efficacy of cetuximab and intensity of EGFR staining in tumours (Cunningham *et al*, 2004; Saltz *et al*, 2004). In addition, response to cetuximab has been observed also in patients with EGFR-negative tumours (Chung *et al*, 2005). These data indicate that EGFR expression by IHC is insufficient to determine candidacy for cetuximab therapy. No reliable markers have so far been characterised to identify patients who will benefit from cetuximab therapy, and only skin reaction has been significantly associated with response and overall survival (OS) (Cunningham *et al*, 2004; Saltz *et al*, 2004). However, recent data suggest that the *EGFR* gene status may predict response to cetuximab (Moroni *et al*, 2005), while *K-Ras* point mutations seem to confer resistance to this drug (Lievre *et al*, 2005; Di Fiore *et al*, 2007). Our study aimed to examine whether molecular determinants such as the *EGFR* gene status and the EGFR downstream cascade members *K-Ras* and PTEN, which are altered in a significant proportion of sporadic CRC independently of the *EGFR* status, may serve as markers in predicting response in patients with mCRC treated with cetuximab.

## PATIENTS AND METHODS

### Patient population and treatment regimens

We analysed 27 consecutive patients, who gave informed consent, with histologically confirmed mCRC at the Institute of Pathology, Locarno, Switzerland. All patients were treated with cetuximab-based regimens at the Oncology Institute of Southern Switzerland, 18 of them treated within clinical trials. All patients had EGFR expression in their primary tumour specimens at IHC.

With the exception of four patients who received cetuximab as frontline therapy, the others had failed at least one prior chemotherapy regimen (Table 1). For those who progressed on irinotecan-based chemotherapy, the MoAb was administered in combination with irinotecan given at the same dose and schedule previously used. Cetuximab was administered at standard loading dose of 400 mg m^−2^ over 2 h, followed by weekly 250 mg m^−2^ over 1 h. Treatment was continued until progressive disease (PD) or toxicity occurred, according to the standard criteria (Therasse *et al*, 2000) or the specific trial guidelines.

### Clinical evaluation and response criteria

The response was assessed every 6 weeks with radiological examination (computerised tomodensitometry or magnetic resonance imaging). The RECIST (Response Evaluation Criteria in Solid Tumors) criteria were adopted for evaluation, and classified into partial response (PR), stable disease (SD) and PD. Patients with SD or PD were defined as nonresponders (Therasse *et al*, 2000). Response to therapy was also evaluated retrospectively by independent radiologists.

### Molecular analyses

Primary tumour specimens were fixed in 10% buffered formalin and embedded in paraffin, and data processing was accomplished at the Institute of Pathology, Locarno, Switzerland. All formalin-fixed paraffin-embedded tumour blocks were reviewed for quality and tumour content, and a single representative tumour block from each case, containing at least 70% of neoplastic cells, was selected for immunohistochemical, cytogenetic and molecular analyses. Genomic DNA was extracted using the QIAamp Mini kit (Qiagen, Chatsworth, CA, USA) according to the manufacturer's instructions.

#### Epidermal growth factor receptor: IHC

Epidermal growth factor receptor protein expression was evaluated using the EGFR PharmDX kit (Dako Cytomation, Glostrup, Denmark) on 3-*μ*m thick tissue sections without knowledge of clinical data or the results of other analyses. The intensity of reaction was classified as score 1+, 2+ or 3+, on the basis of the percentage of positive cells and the intensity of staining, according to the manufacturer's instructions (<5%, 5–50% and >50% of cells, respectively). As controls, we used those included in the kit.

#### Microsatellite instability

The status of microsatellite instability (MSI) was assessed by the analysis of the microsatellite loci included in the panel of Bethesda (*BAT25*, *BAT26*, *D2S123*, *D5S346*, *D17S250*), as reported previously (Frattini *et al*, 2004). Microsatellite instability was confirmed by the presence of an additional peak in tumour sample in comparison with normal paired tissue.

#### Epidermal growth factor receptor: fluorescent *in situ* hybridization

Epidermal growth factor receptor gene status evaluation was performed by fluorescent *in situ* hybridization (FISH) on 3-*μ*m thick tissue sections. Tissue sections were treated using Paraffin Pretreatment kit II (Vysis, Downer's Grove, IL, USA) according to the manufacturer's instructions. Dual-colour FISH assay was performed using LSI EGFR/CEP7 probes (Vysis). The LSI EGFR probe is labelled in SpectrumOrange and covers an approximately 300 kb region that contains the entire *EGFR* gene at 7p12. The CEP7 probe, labelled in SpectrumGreen, hybridises to the alpha satellite DNA located at the centromere of chromosome 7 (7p11.1–q11.1). Target sections and probe were co-denatured at 75°C for 5 min and allowed to hybridise overnight at 37°C. Post-hybridisation stringency wash was carried out in water bath at 72°C for 5 min. After washing twice and drying at room temperature for 10 min, slides were mounted with 4′6-diamidino-2-phenylindole (DAPI II; Vysis). Fluorescent *in situ* hybridization signals were evaluated with a Zeiss Axioscope equipped with single and triple band pass filters. Image for documentation were captured using AxioCam camera and processed using the AxioVision system. Patients showing two chromosome 7 in the vast majority of cells were classified as eusomic. Patients with an aberrant number of chromosome 7, defined as more than 4 in at least 50% of cells, were classified as markedly polysomic. Patients with a ratio more than 3 between *EGFR* gene and chromosome 7 centromere signals in at least 10% of cells were classified as having EGFR gene amplification.

#### *K-Ras* mutational status: sequencing

We searched for *K-Ras* point mutations in codons 12 and 13, two hotspots that include more than 95% of mutations in this gene, as already reported (Frattini *et al*, 2004). All samples were subjected to automated sequencing by ABI PRISM 3100 (Applied Biosystems, Foster City, CA, USA) and analysed with Chromas software. Each sequence reaction was performed at least twice, starting from independent PCR reactions. In each case, the detected mutation was confirmed in the sequence as sense and antisense strands.

#### PTEN expression: IHC

PTEN protein expression status by IHC on 3-*μ*m tissue sections was performed and evaluated according to the literature (Frattini *et al*, 2005; Saal *et al*, 2005). The Anti-PTEN Ab-2 (Neomarkers, Fremont, CA, USA) was applied at 1 : 50 dilution. PTEN protein expression was detected mainly at cytoplasmic level, although occasional nuclear positivity was present. We considered PTEN negative tumours those showing a dramatical reduction or absence of immunostaining in at least 50% of cells, as compared with the internal control. The evaluations were performed without knowledge of clinical data or the results of other analyses.

### Statistical considerations

The objective tumour response was the end point of our exploratory study. The two-tailed Fisher's exact test was used to calculate *P*-value for association between the gene alterations and response to cetuximab. The level of significance was set at *P*=0.05.

The OS time was calculated as the period from the first day of cetuximab treatment until death from any cause, or the date of the last follow-up.

## RESULTS

A total of 27 patients were analysed, including nine women and 18 men, with a median age of 67 years (range, 29–84 years). Colon and rectal cancers were diagnosed in 19 and eight patients, respectively, and synchronous metastases were found in 19 patients (70%). Cetuximab was administered in combination with chemotherapy as upfront therapy in four cases, as second line in seven cases, as third line in 13 patients and as fourth line in three cases. Ten patients (37%) achieved PR after cetuximab-based therapy, and the median duration of response was 21 months (range, 8–48 months). Characteristics and response by treatment are summarised in Table 1.

### Microsatellite instability

Using the Bethesda panel, none of tumours showed MSI (data not shown).

### Epidermal growth factor receptor: IHC and FISH

**Figure 1:**
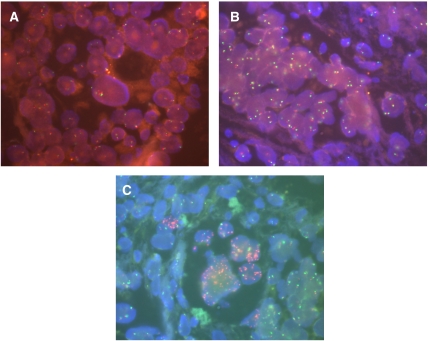
Epidermal growth factor receptor gene status evaluated by FISH in metastatic colorectal cancers. (**A**) Patient showing eusomy of chromosome 7. (**B**) Patient with marked polysomy on chromosome 7. (**C**) Patient with *EGFR* gene amplification in at least 10% of tumoral cells.

Table 2 summarises the immunohistochemical, cytogenetic and molecular features. All patients had EGFR-positive tumours at IHC, five cases (19%) were classified as score 1+, nine cases (33%) as score 2+ and 13 (48%) as score 3+. By FISH, three (11%) patients showed eusomy, 16 (59%) patients were highly polysomic on chromosome 7 and eight (30%) patients showed *EGFR* amplification (Figure 1). Five patients (Table 2) presented rare cells with *EGFR* amplification associated with a large majority (>50%) of cells with marked polysomy, and consequently they were classified as highly polysomic. We did not find any significant correlation by comparing the EGFR protein expression (by IHC) and its gene status (by FISH). In fact, those with eusomy showed either score 1+ (66%) or score 3+ staining (33%). Polysomy correlated with score 1+ in 6%, score 2+ in 44% and score 3+ in 50%. Patients who had gene amplification were equally distributed, with prevalence in favour of score 3+ (score 1+, 2+ and 3+: 25, 25 and 50%, respectively).

### *K-Ras* status

Seventeen patients did not show *K-Ras* mutations. Point mutations were found in 37% of cases, in seven patients on codon 12 and in three patients on codon 13 (Table 2). Mutations on codon 12 predominantly involved the second base, with prevalence of the *GaT* mutation (*GGT* → *GaT*, Gly → Asp, G12D), which was observed in four cases. In one case, the mutation on codon 12 involved the first base (*GGT* → *tGT*, Gly → Cys, G12C). No *GtT* alteration on codon 12 was found, which represents one of the most frequent mutations observed in sporadic CRC. The mutations found on codon 13 corresponded to the transition *G → A* to the second base of the codon (*GGC* → *GaC*, Gly → Asp, G13D).

### PTEN: IHC

**Figure 2:**
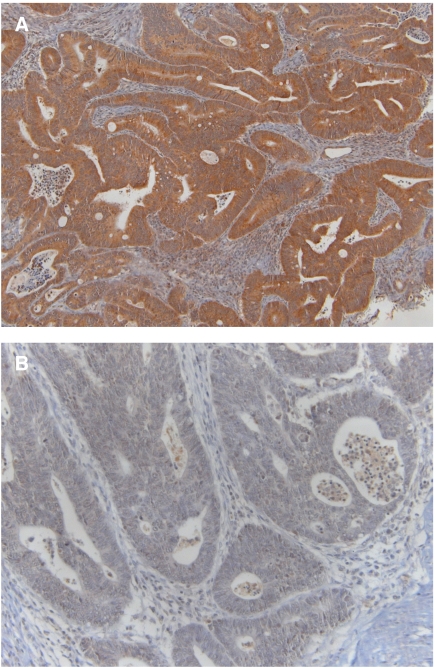
PTEN protein expression by immunohistochemistry in metastatic colorectal cancers. (**A**) Patient showing normal PTEN expression. (**B**) Patient with absent PTEN expression.

Normal PTEN expression was documented in 16 (59%) patients, while loss of PTEN protein expression was found in 11 patients (Figure 2).

### Molecular markers and response to cetuximab

Eusomic patients did not respond to cetuximab-based therapy. An objective response was observed in four out 16 (25%) patients with polysomy, while six out eight (75%) patients with *EGFR* amplification were considered as responders (Table 3). The correlation of the EGFR gene status and response reached statistical significance (*P*<0.05).

Only one out 10 (10%) patients with mutated *K-Ras* experienced a response to cetuximab-based therapy, while nine out of 17 (53%) patients with wild-type sequence had a benefit from therapy. The correlation of the *K-Ras* status and response reached statistical significance (*P*<0.05) (Table 3). The kind of mutation observed did not influence the response.

As regards to PTEN, 10 of 16 (62.5%) patients with intact protein expression had an objective response to cetuximab-based therapy (Table 3). In contrast, none of the 11 patients with loss of PTEN protein expression had an objective benefit from the MoAb (*P*<0.001).

## DISCUSSION

The Food and Drug Administration (FDA) approved cetuximab in 2004 for treatment of mCRC in combination with irinotecan, as well as in monotherapy in patients intolerant to irinotecan. However, only a minority of patients respond to cetuximab-based therapy, and there are currently no molecular markers able to identify patients who will benefit from this therapeutic approach. A better understanding of molecular mechanisms that may predict resistance or response to cetuximab is therefore urgently needed.

Similarly to previous reports (Cunningham *et al*, 2004; Saltz *et al*, 2004), we observed no correlation between the intensity of EGFR expression as detected by IHC and response to cetuximab-based therapy. In the present cohort, eusomic patients who did not respond to cetuximab were actually classified either score 1+ (66%) or score 3+ (33%). These results are in agreement with those from two small trials, showing that EGFR evaluation using IHC is misleading in predicting response to the MoAb. Moreover, in a retrospective series of 16 chemo-refractory patients not expressing EGFR, cetuximab was shown to produce an RR of 25% (Chung *et al*, 2005). In addition, two PR were seen in nine patients with EGFR-negative tumours enrolled in a phase II study of single agent cetuximab (Lenz *et al*, 2005). It has been shown that the choice of fixative and storage time of tumour tissue, (Atkins *et al*, 2004) the choice of primary antibody and scoring system (Kersting *et al*, 2006), and the lack of standardised criteria for evaluation (Langner *et al*, 2004) all represent potential pitfalls and have a substantial impact on determination of EGFR immunoreactivity. It is therefore highly questionable whether mCRC patients should be selected for cetuximab-based therapy only on EGFR reactivity by IHC.

Our results indicate that both high polysomy on chromosome 7 and *EGFR* gene amplification appear to be a pre-requisite for response to cetuximab (observed in 25 and 75% of cases, respectively). Supporting this hypothesis, three eusomic patients had no benefit from the MoAb. Our findings are consistent with those reported (Moroni *et al*, 2005; Lievre *et al*, 2005). Of note, in one of these, patients with trisomy were defined as polysomic and benefited from the drug in 89% of cases (Moroni *et al*, 2005). In our series, however, 12 cases with high polysomy on chromosome 7 and two cases with *EGFR* gene amplification did not benefit from cetuximab. Consequently, the evaluation of the *EGFR* gene status appears to be insufficient to predict response to the MoAb.

In the present cohort, the majority (90%) of patients with mutated *K-Ras* did not benefit from cetuximab. Similar results have been reported in two additional trials, whereas in another study a not significant trend was observed. If we pool our data with those of these three studies (Moroni *et al*, 2005; Lievre *et al*, 2006; Di Fiore *et al*, 2007), the difference between *K-Ras* mutated and *K-Ras* wild-type sequence patients as regards to cetuximab treatment is highly significant, indicating that the assessment of *K-Ras* mutations in mCRC plays a fundamental role in predicting cetuximab efficacy. As regards to the type of *K-Ras* mutation, the mostly observed alterations occurred on codon 12 (70%), against 30% on codon 13. These findings are consistent with previous reports (Frattini *et al*, 2004). Interestingly, besides the expected occurrence of *GaT* mutation, no alterations of *GtT* (the other most frequently observed *K-Ras* mutation in sporadic CRC) (Frattini *et al*, 2004) were found on codon 12. The present data reinforce, therefore, the knowledge that the *GtT* mutation typically correlates with an indolent clinical course, and seldom occurs in mCRC (Sarli *et al*, 2004).

Other factors besides the *EGFR* gene status and *K-Ras* mutations are likely to be involved in mechanisms of resistance to cetuximab. The EGFR signal activation leads not only to downstream effects on Ras-MAP kinase pathway, but also regulates the PTEN-PI3K-Akt cascade. The loss of expression of PTEN protein has been observed in 30% of sporadic CRC (Thomas and Grandis, 2004). No data on PTEN protein expression and correlation with response to cetuximab in mCRC have been reported yet. We demonstrate that loss of PTEN protein expression may be a useful marker in predicting response to cetuximab. In fact, none of the 11 patients with loss of PTEN expression did benefit from the treatment with MoAb, while a response was observed in 10 out of 16 patients with intact PTEN expression. The six patients with intact PTEN expression who did not benefit from cetuximab had *K-Ras* mutation in four cases, eusomy in one case and both in one case: all these factors have been shown to predict resistance to cetuximab.

The effect of PTEN expression on cetuximab response is similar to the one observed in trastuzumab-treated breast cancer patients (Pandolfi, 2004), supporting the concept that PTEN expression plays a fundamental role in predicting the response to drugs against HER family members.

The population of the present study is comparable with those of previous related studies in terms of included patients, and it is homogeneous since it encompasses unselected patients native from an isolated geographic area, all evaluated and treated in one institution. None of the patients were found to exhibit MSI, which suggests that tumour development in our patients followed the same pathway (Fearon and Vogelstein, 1990). Actually, the rate of PR (37%) is surprisingly high when compared with published data of pivotal trials including cetuximab (Cunningham *et al*, 2004). On the other hand, our cohort is characterised by high frequency of polysomy and *EGFR* gene amplification. We can therefore speculate that environmental and lifestyle factors might lead both to frequent cell division deregulation (as deductible by the observed high rate of polysomy) and to *EGFR* gene amplification. Although the clinical course might be more aggressive, this particular constellation makes perhaps a targeted approach more effective than expected.

The possibility that the present findings are related to the response to the previous chemotherapy regimen rather than cetuximab sensitivity or resistance may raise questions on the validity of our as well as previous results. This hypothesis, however, is unlikely since all patients included in this study were refractory to previous chemotherapeutic treatment, and drugs, such as fluoropirimidines, oxaliplatin and irinotecan, act against thymidylate synthase and topoisomerase I and not against the EGFR signalling pathway.

**Figure 3:**
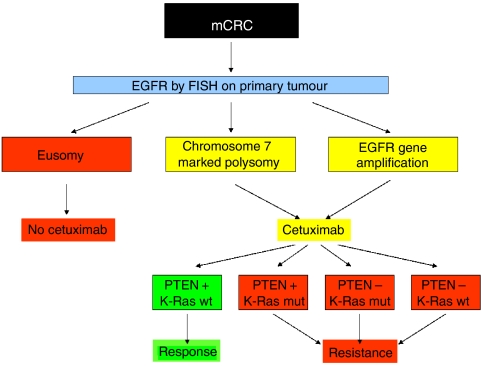
Algorithm in predicting response to cetuximab according to the *EGFR* and *K-Ras* status, and PTEN protein expression.

Overall, our findings allow to propose an algorithm in order to possibly select patient for cetuximab therapy (Figure 3). Those presenting with eusomy on chromosome 7 are more likely to be refractory to the MoAb, while patients with high polysomy or *EGFR* gene amplification should be considered for cetuximab therapy. A benefit from the MoAb may be expected in patients presenting with wild-type *K-Ras* and intact PTEN expression. Only one patient escaped to this algorithm, in that he responded to cetuximab in presence of *K-Ras* mutation. A possible explanation could be that in our series all molecular analysis have been performed on primary CRC, and the gene profile on primary tumour and metastasis might differ, as previously reported (Scartozzi *et al*, 2004).

In conclusion, our results indicate that different downstream proteins of the EGFR cascade have a deep effect on response to cetuximab. In particular, this is the first report on the predictive role of the expression of PTEN protein in mCRC. These data, which need to be validated in large prospective clinical trials, might represent a valid platform for oncologists in selecting patients for cetuximab-based therapy, with evident clinical and economical consequences.

## Figures and Tables

**Table 1:** Patient's characteristics and response by treatment

**Patients**	**Sex**	**Age (years)**	**Previous therapies**	**Cetuximab line**	**Regimen**	**Best response**	**Duration of response (weeks)**
1	F	65	FOLFOX	2nd	CPT11-b/r+cetuximab (CT)	PR	8
2	M	67	FOLFOX; CPT11	3rd	CPT11-b/r+cetuximab	PD	NA
3	M	60	FOLFOX; CAP; FOLFIRI	4th	CPT11-b/r+cetuximab (CT)	PD	NA
4	M	82	CAPOX	2nd	CPT11-b/r+cetuximab (CT)	PD	NA
5	M	78	CAP; FOLFOX; FOLFIRI	4th	CPT11-b/r+cetuximab (CT)	PD	NA
6	F	63	CPT11	2nd	CPT11-b/r+cetuximab (CT)	PD	NA
7	M	72	CPT11	2nd	CPT11-b/r+cetuximab (CT)	PD	NA
8	F	59	FOLFIRI	2nd	CPT11-b/r+cetuximab (CT)	PR	48
9	M	69	FOLFIRI	2nd	CPT11-b/r+cetuximab (CT)	PR	16
10	M	59	FOLFOX; FOLFIRI	3rd	CPT11-b/r+cetuximab	PR	48
11	M	67	FOLFOX; CPT11	3rd	CPT11-b/r+cetuximab (CT)	PD	NA
12	M	69	FOLFOX	2nd	CPT11-b/r+cetuximab (CT)	PD	NA
13	M	79	CAPOX; CPT11	3rd	CPT11-b/r+cetuximab	PR	13
14	M	65	None	1st	CAPOX+cetuximab (CT)	PD	NA
15	F	75	CAPOX; FOLFIRI	3rd	CPT11-b/r+cetuximab	PD	NA
16	M	64	5FU; CAPOX; FOLFIRI	4th	CPT11-b/r+cetuximab (CT)	PR	12
17	M	72	FOLFOX; CAPIRI	3rd	CPT11-b/r+cetuximab (CT)	PR	28
18	F	72	5FU/LV; CPT11	3rd	CPT11-b/r+cetuximab (CT)	SD	8
19	M	66	CAPIRI; CPT11	3rd	CPT11-b/r+cetuximab	PD	NA
20	F	63	None	1st	CAPOX+cetuximab (CT)	PR	8
21	F	84	CAP; CPT11	3rd	CPT11-b/r+cetuximab	PD	NA
22	M	59	CAPOX/BV; FOLFIRI	3rd	CPT11-b/r+cetuximab	SD	1
23	M	78	FOLFOX; CPT11	3rd	CPT11-b/r+cetuximab	PD	NA
24	F	29	FOLFOX; FOLFIRI	3rd	CPT11-b/r+cetuximab (CT)	SD	16
25	M	58	FOLFOX; CPT11	3rd	CPT11-b/r+cetuximab	PR	16
26	M	70	None	1st	CAPOX+cetuximab (CT)	PR	13
27	F	44	None	1st	CAPOX+cetuximab (CT)	PD	NA

Abbreviations: BV, bevacizumab; CAP, capecitabine; CAPIRI, irinotecan and CAP; CAPOX, oxaliplatin and CAP; CPT11-b/r, irinotecan-based regimen; CT, clinical trial; F, female; 5FU, fluorouracil; FOLFIRI, irinotecan, 5FU and folinic acid; M, male; FOLFOX, oxaliplatin, 5FU and folinic acid; NA, not applicable; PD, progressive disease; PR, partial response; SD, stable disease.

**Table 2:** Immunohistochemical, cytogenetic and molecular data

**Patients**	**EGFR: IHC**	***EGFR*: FISH**	***K-Ras*: status**	**PTEN: IHC**
1	1+	A	WT	Pos
2	2+	A	13GaC	Neg
3	3+	P	12GcT	Pos
4	1+	P	WT	Neg
5	3+	P	12GcT	Pos
6	2+	P	12GaT	Neg
7	2+	P	WT	Neg
8	3+	A	WT	Pos
9	3+	A	WT	Pos
10	2+	P^*^	WT	Pos
11	1+	E	13GaC	Neg
12	3+	E	12GaT	Pos
13	3+	A	WT	Pos
14	3+	P^*^	WT	Neg
15	2+	A	12tGT	Neg
16	2+	P	WT	Pos
17	1+	A	WT	Pos
18	2+	P^*^	12GaT	Pos
19	3+	P^*^	WT	Neg
20	3+	P	13GaC	Pos
21	3+	P	12GaT	Pos
22	1+	E	WT	Pos
23	3+	P	WT	Neg
24	3+	P	WT	Neg
25	3+	A	WT	Pos
26	2+	P^*^	WT	Pos
27	2+	P	WT	Neg
Total	5/27 (19%) score 1+	8/27 (30%) A	17/27 (63%) WT	16/27=59% Pos
	9/27 (33%) score 2+	16/27 (59%) P	10/27 (37%) Mutated	11/27=42% Neg
	13/27 (48%) score 3+	3/27 (11%) E		

Abbreviations: A, *EGFR* gene amplification; P, chromosome 7 polysomy; P^*^, polysomy with rare cells showing *EGFR* amplification; E, eusomy; WT, wild type; Pos, positive; Neg, negative.

**Table 3:** *EGFR* and *K-Ras* gene status, PTEN protein expression: correlation with clinical response to cetuximab

	***EGFR* (FISH)**			***K-Ras***		**PTEN**	
	**E**	**P**	**A**	**WT**	**Mut**	**Pos**	**Neg**
Clinical response to PR	0	4	6	9	1	10	0
Cetuximab NR	3	12	2	8	9	6	11
Fisher exact test	*P*<0.05			*P*<0.05		*P*<0.001	

Abbreviations: PR, partial response; NR, nonresponder; E, eusomy; P, chromosome 7 polysomy; A, *EGFR* gene amplification; WT, wild type; Mut, mutated; Pos, positive; Neg, negative.
